# The Effect of Smear Layer on Antimicrobial Efficacy of Three Root Canal Irrigants 

**DOI:** 10.7508/iej.2015.03.007

**Published:** 2015-07-01

**Authors:** Nazanin Zargar, Omid Dianat, Mohammad Asnaashari, Mojtaba Ganjali, Saeede Zadsirjan

**Affiliations:** a *Department of Endodontics, Dental School, Shahid Beheshti University of Medical Sciences, Tehran, Iran; *; b * Iranian Center for Endodontic Research (ICER), Research Institute of Dental Sciences, Department of Endodontics, Dental School, Shahid Beheshti University of Medical Sciences, Tehran, Iran*

**Keywords:** *Actinomyces israelii*, *Candida albicans*, Chlorhexidine, Povidone-Iodine, Smear Layer, Sodium Hypochlorite

## Abstract

**Introduction:**

One of the main goals of endodontic treatment is to decrease the harboring bacteria within the root canal system and dentinal tubules. This experimental study attempted to investigate the antibacterial efficacy of three root canal irrigants in the presence and absence of smear layer (SL).

**Methods and Materials:**

A total of 210 sound extracted human single-rooted teeth were prepared. After creating the SL and its removal in half of the samples, they were infected with *Candida albicans* (*C. albicans*) and *Actinomyces israelii (A. israelii)*. A total of 180 specimen were used to assess the antimicrobial efficacy of the three irrigants in presence and absence of SL, 24 specimen were placed in the positive and negative controls, 2 samples were utilized for scanning electron microscopy (SEM) analysis and 2 were used for Gram staining. Then, they were exposed to irrigants including 2.61% sodium hypochlorite (NaOCl), 0.2% chlorhexidine gluconate (CHX) and 1% povidone-iodine (PI) for 5, 30 and 60 min. Presence/absence of test microorganisms was determined by incubation of specimens in test tubes containing brain-heart infusion (BHI) broth and then measuring the colony forming units (CFU) on BHI agar. A cumulative logistic model was used to analyze the ordinal response.

**Results:**

The 2.61% solution of NaOCl was significantly more effective than 0.2% CHX and the latter was more efficient than 1% PI for decreasing fungal and microbial infection of dentinal tubules in the presence and absence of SL.

**Conclusion:**

The presence of smear layer decreased the efficacy of antimicrobial irrigants. The minimum time required for elimination of fungal/microbial infection was 30 min.

## Introduction

Microorganisms are the most important etiologic factors in development of pulp and periapical pathosis [1]. Complete elimination of pathogenic microorganisms from the complex root canal systems (RCS) and particularly dentinal tubules is a challenge in endodontic treatment. The most important cause of endodontic failure is the residual microorganisms harbored within the RCS and hard-to-reach areas [2]. Use of endodontic irrigants during root canal preparation decreases the number of different microorganisms [3].

Smear layer (SL) is a physical barrier that decreases the penetration of disinfecting agents into the dentinal tubules and consequently, their efficacy. Moreover, after canal preparation, entrapped intra-tubular bacteria may be entombed by the SL [4]. 

There are many studies with controversial results comparing the antimicrobial properties of different intracanal disinfecting solutions such as sodium hypochlorite (NaOCl), chlorhexidine gluconate (CHX) and povidone-iodine (PI) against various pathogenic microorganisms. No consensus has been reached regarding the superiority of a specific type of irrigant, the optimal concentration or duration of exposure for achieving the maximum efficacy [5].

Ideal endodontic irrigants should have bacteriostatic properties and must be capable of dissolving the pulp remnants and the SL. At the same time, they must have low toxicity [6, 7]. Many previous studies have demonstrated the optimal efficacy of endodontic disinfecting solutions in SL removal from the intact dentinal surfaces [4, 8, 9]. However, no evidence is available regarding the superiority of a specific irrigant with greater antimicrobial efficacy in presence of SL. Moreover, the mechanism of action against infected dentin in the presence of SL has yet to be clearly understood.

*Actinomyces israelii (A. israelii) *and *Candida albicans (C. albicans)* are among the microbial strains that may remain in the RCS even after disinfection. They are coaggregation partners [10, 11] and considered amongst the treatment-resistant microorganisms that are commonly involved in treatment failure [11]. 

The purpose of this study was to compare the antimicrobial effects of three endodontic irrigants including 2.61% NaOCl, 0.2% CHX and 1% PI, on *C. albicans* and *A. israelii* cultured in dentinal tubules in the presence or the absence of SL after different time durations.

## Materials and Methods

This *in vitro* study was conducted on 210 sound, single-rooted, single-canal human teeth with non-calcified canals extracted for periodontal or orthodontic reasons. These teeth were free from caries or coronal restoration and had straight roots and mature apices. They were stored in 2% thymol solution. 

***Preparation of specimens*** *:*

For standard preparation of root canals, the teeth were immersed in 5.25% NaOCl solution for 5 min followed by immersion for another 5 min in distilled water. The crowns were cut at the cemento-enamel junction (CEJ) and the apical end was also cut using a diamond fissure bur (Diatech Dental AG, Heerbrugg, Switzerland) installed on high-speed handpiece (W&H, Buernoos, Austria) so that the length of residual root segment measured 8 mm. The cementum covering the external root surface was removed using a diamond fissure bur in order to enhance penetration of microorganisms into dentinal tubules and enable controlled tubular infection. 

Then the samples were divided into two groups (*n*=104). In group 1 (SL+), specimens were prepared with #2 to 5 Gates Glidden drills (Dentsply Maillefer, Ballaigues, Switzerland)

while in group 2 (SL-) preparation was done with #2 to 6 of the same drills. All specimens were immersed in 17% ethylenediaminetetraacetic acid (EDTA) solution at a pH of 7.8 in an ultrasonic bath (Biosonic UC 300, Coltane Whaledent Inc., NY, USA) for 4 min followed by immersion in 5.25% NaOCl for 4 min. Then in group 1 (SL+), the SL was created by widening the canal with # 6 Gates Glidden drill. To this point both groups had similar preparation sizes.

**Figure1:**
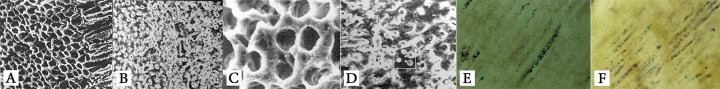
SEM images showing removal *(A)* and presence *(B)* of smear layer, bacterial growth in specimens without *(C)* and with *(D)* smear layer, microbial growth controlled by Gram staining in specimen without *(E)* and with *(F)* smear layer (400× magnification

The specimens were rinsed with sterile distilled water for 10 min. Presence and absence of SL was confirmed by assessment of 2 specimens under scanning electron microscopy, SEM (Zeiss DSM 940A, Carl Zeiss Inc., Germany) (Figures 1A and 1B). Then all samples from both SL+ and SL- groups were divided for comparing the efficacy of 2.61% NaOCl, 0.2% CHX (Shahrdaru, Tehran, Iran) and 1% PI (Betadine, Ramin Laboratory, Tehran, Iran) after 5, 30 and 60 min. Also, a total of 24 teeth (12 from each SL+ and SL- groups) were considered as the positive and negative controls and 2 specimens were used to ensure bacterial growth under SEM and Gram staining.

Specimens were cold sterilized by means of ethylene oxide gas and were then individually transferred to screw-top vials containing brain-heart infusion (BHI) broth (Biolife, Milan, Italy) under aseptic conditions.

***Bacterial inoculation:***

Specimens were inoculated with a mixed microbial suspension containing *C. albicans* and *A. israelii* comparable to No. 0.5 McFarland standard with approximately 1.5×10^8^ CFU/mL.

Fresh, 24-h microbial suspension was transferred to each flask in equal volumes of 5 cc. Sterilized specimens of both groups were aseptically placed in the flasks of culture medium suspensions. All flasks were stored under aerobic conditions at 37^°^C. After 24 h, 2 cc of fetal calf serum (FCS) (BaharAfshan Co., Tehran, Iran) was added to each flask. 

**Table 1:** Cumulative logistic model of the main effects and second order effects for microbial infection (confidence interval=95%)

**Variable **	**Variable level**	**Estimate**	**Standard Error**	***P-value***	**Odds Ratio**	**Interval for odds ratio**
**Smear**	Present (baseline)	-2.880	1.037	0.005	0.0561	(0.007, 0.428)
	Absent					
**Time**	60 min (baseline)					
	30 min	-2.776	1.176	0.018	0.062	(0.006, 0.624)
	5 min	-3.210	1.077	0.003	0.040	(0.005, 0.333)
**Irrigant**	PI (baseline)					
	NaOCl	-2.776	1.176	0.018	0.062	(0.006, 0.624)
	CHX	-3.210	1.077	0.003	0.040	(0.005, 0.333)
**Smear/Time**	No smear/5 min	2.224	1.036	0.032	9.244	(1.213, 70.426)
	No smear/30 min	1.322	1.040	0.204	3.751	(0.489, 28.801)
**Irrignt/Time**	NaOCl/5 min	1.204	1.323	0.363	3.333	(0.249, 44.571)
	CHX/5 min	1.083	1.146	0.345	2.953	(0.312, 27.915)
	NaOCl/30 min	2.062	1.319	0.118	7.862	(0.593, 104.298)
	CHX/30 min	2.662	1.128	0.018	14.325	(1.570, 130.697)
**Smear/Irrigant**	No smear/NaOCl	-1.294	1.005	0.198	0.274	(0.038, 1.966)
	No smear/CHX	0.981	0.878	0.264	2.667	(0.477, 14.908)

Specimens were incubated at 37°C under aerobic conditions for 14 days. Proliferation of pure microorganisms was controlled by Gram staining of the broth suspension. After completion of the incubation periods, the broth was exchanged with 3 cc of 0.1 M Sorensen's phosphate buffer (SPB) solution (pH=7.4); each tube was then put on a rotator for 40 sec. Afterwards the SPB was removed from the tubes. This procedure was repeated for three times for each tube to remove the excess culture medium and the bacteria floating on the surface of specimen. At this step, two specimens (one from each SL+ and SL- group) were prepared for SEM analysis (Figures 1C and D). Two other specimens (from each group) were prepared for Gram staining to ensure presence and proliferation of microorganisms inside the dentinal tubules (Figures 1E and F). Next, the external surfaces of specimens were dried with sterile gauze and coated with nail varnish.

***Exposure to irrigants:***

Specimens were aseptically transferred to sterile, screw-top flasks containing 3 cc of each irrigant (2.61% NaOCl, 0.2% CHX and 1% PI). Next, the flasks were incubated for 5, 30 and 60 min at 37^°^C temperature under aerobic conditions (*n*=30). Positive control specimens were subjected to sterile 0.9% saline solution and the samples of negative control group were not exposed to any irrigant. After completion of incubation period, the irrigants were extracted from the flasks and each specimen was rinsed with 3 cc of 0.1 M SPB on a rotator for three times. Specimens were then transferred to screw-top flasks containing sterile BHI broth and were incubated at 37^°^C for 24 h.

***Growth determination:***

The flasks were thoroughly evaluated, the degree of turbidity was compared with McFarland standard and recorded. Specimens were maintained in incubator and their turbidity was evaluated again after 7 days. The new and old specimens were separately cultured in BHI agar to count the *C. albicans* and *A. israelii* colonies. During the study period, the growth of test microorganisms was checked by Gram staining. Specimens that did not show turbidity and remained clear for 2 weeks were considered as negative culture and their McFarland concentration and number of CFU were recorded as zero. These specimens were inoculated with 0.01 mL of fresh microbial suspension containing *C. albicans* and *A. israelii* in order to ensure that the culture medium in test tubes did not cause microbial inhibition via possible, accidental transmission of irrigants. According to the classification by Kuruvilla and Kamath [12], CFU<10^3^, 10^3^<CFU<10^5^ and CFU≥10^5 ^was considered low, moderate and high, respectively. Agar diffusion test was used to assess the growth inhibition zones and confirm the antimicrobial properties of irrigants.

***Statistical analysis:***

An ordinal response was considered by categorizing count data and the cumulative logistic model (proportional odds model) was used to analyze the ordinal response. Odds ratios were used to compare the antimicrobial effects of the three endodontic irrigants. The SPSS software (SPSS version 22, SPSS, Chicago, IL, USA) was used to obtain the results.

## Results

According to cumulative logistic model, the predicted probability of low CFU counting (CFU<10^3^) was more in the absence of SL (*P*=0.005). The same can be inferred using NaOCl and CHX (*P*=0.018 and 0.003, respectively). On the other hand, the predicted probability of low values of microbial infection, was less with 30-min CHX samples (*P*=0.018) and in the absence of SL in 5-min samples (*P*=0.032).

According to odds ratios**, **the odds of having low values of microbial infection was more for NaOCl specimens compared to CHX samples in both 5-min and 30-min durations (odds ratio=1.644 and 3.446, respectively). In the absence of SL, the odds of having low-value microbial infection was similar for both irrigants in 60-min (odds ratio=1).

In the presence of SL, the odds of having low values of microbial infection was more for NaOCl compared to CHX at both time intervals of 5 and 30-min (odds ratio=1.641 and=3.448, respectively); however this ratio was similar (odds ratios=1) for both irrigants in 60-min samples.

CHX was better than PI even with longer time intervals (odds ratios=10.280) (Table 1).

## Discussion

The purpose of this study was to compare the antimicrobial effect of 2.61% NaOCl, 0.2% CHX and 1% PI on dentin models infected with* C. albicans* and *A. israelii* in the presence or the absence of SL in different time intervals (5, 30 and 60 min).

NaOCl had the highest efficacy against test microorganisms cultured in dentinal tubules followed by 0.2% CHX and 1% PI.

The greater efficacy of 2.61% NaOCl compared to 0.2% CHX in SL- samples can be due to the strong oxidative potential of NaOCl [13]. The antibacterial efficacy of NaOCl is *via* irreversible oxidation of sulfhydryl group of bacterial enzymes, its destructive effect on bacterial DNA and other membrane-related activities [14, 15]. Due to its alkalinity, NaOCl has strong ionic power. It is capable of dissolving vital and non-vital tissues and has proteolytic properties enhancing its flow [16]. Due to these reasons, it is still the most commonly used endodontic irrigant [17]. On the other hand, CHX has high molecular weight and can barely penetrate into the tubules [18]. Although 0.2% CHX has high antimicrobial activity, it cannot dissolve tissues [19]. 

The higher antimicrobial efficacy of NaOCl compared to CHX in this study is in accordance with findings of Siqueira *et al.* [20], Ørstavik *et al.* [21] and Wang *et al.* [4]. However, Ørstavik *et al.* [21], Şen *et al.* [22], Heling *et al.* [23] and Siqueira *et al.* [24] reported comparable antimicrobial efficacy of these two irrigants; although, CHX was more effective on superficial layers.

PI is an excellent antiseptic that gradually releases iodine and is well tolerated by mucosa. It can penetrate into the dentinal tubules and exert its antimicrobial effects, but its antimicrobial efficacy depends on the type of microorganism. This finding is somehow in accordance with the results reported by of Ringel *et al*. [25], Delany *et al.* [26], Ohara *et al.* [27], Shurrab [28] and Abdullah *et al.* [29].

These differences may be attributed to study conditions, type of microorganisms, difference in percentage (concentration) of irrigants used and methodology of studies. The current study was conducted on human dentin that better simulates the clinical setting. Also, the selected microorganisms were amongst the treatment-resistant strains [11, 30, 31].

The 5-min antimicrobial efficacy of NaOCl and CHX is different [32]. The minimum time required for the efficacy of a given antimicrobial agents against test microorganisms is 30 min; which is in line with the results reported by Sen *et al.* [22] who assessed the antimicrobial activity of different concentrations of NaOCl and 0.12% CHX against *C. albicans*. The relatively longer time period in their study is probably due to the lower concentration of CHX (0.12%). According to Chau *et al.* [33], the antibacterial activity of NaOCl increases with treatment time. However, Retamozo *et al.* [1] demonstrated that a higher concentration of NaOCl (5.25%) is required to eliminate *E. faecalis* in the absence of SL during 40 min; this finding indicates that some microorganisms are more resistant to antimicrobial agents. Oncag *et al.* [34] compared the antibacterial properties of 5.25% NaOCl, 2% CHX and 0.2% CHX plus 0.2% cetrimide during 5-min and 48-h intervals against *E. faecalis*. They found that 0.2% cetrimide and 2% CHX were more effective on *E. faecalis* than 5.25% NaOCl at both time points.

According to the present study, the effect of SL is significant when NaOCl is used as the disinfecting agent which confirms the results of Wang *et al.* [4]. In our study, no significant difference was noted in the efficacy of CHX between the SL+ and SL- specimens; the greater effect of these solutions on superficial layers and absorption of CHX by the acidic proteins, can be the reason. The SL on deep dentin has greater organic content than the superficial dentin due to the higher number of odontoblastic processes or greater amounts of proteoglycans covering the tubules [35]. CHX is a cation well absorbed by these negatively charged proteins. Thus, it penetrates into the SL and exerts its antimicrobial effect. Moreover, CHX has sustained release; therefore, its antimicrobial efficacy continues over time as the SL is gradually dissolved [36]. 

The importance of tubular patency for effective disinfection of the canal was emphasized by Ørstavik *et al.* [21]. The SL has a significant effect on water diffusion into dentin layers; it is responsible for 86% of resistance against water flow and its removal, dentin permeability increases by 5 to 6 times for diffusion and by 25 to 36 times for filtration. By removing the SL, the tubular diffusion surface increases by 9.7% and dentin permeability increases by 5 times [35]. 

The presence of SL, interferes with perfect adaptation of the root canal filling material with the root canal walls. However, some researchers are against SL removal; stating that the presence of SL prevents bacterial adhesion and colonization of dentinal matrix and inhibits root canal reinfection [37]. According to Love *et al.* [38] the SL acts as a protective barrier and if removed, there would be no physical barrier against bacterial penetration into the dentinal tubules.

The limitation of our study was evaluation of regional disinfection in tubules, although in this method the probability of infecting the specimens with other microorganisms except test microorganisms decreased. The present study may be criticized in that we used low concentration of CHX that was similar to the other experimental studies and has lower toxicity. Conducting further studies with larger sample sizes and with different concentrations of test materials is recommended.

## Conclusion

The presence of smear layer has a negative impact on the efficacy of antimicrobial irrigants. In the presence or absence of smear layer, antimicrobial activity of NaOCl is better than CHX in periods less than 60 min. However, at longer time periods, the antimicrobial efficacy of NaOCl is similar to CHX.
